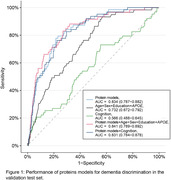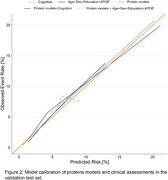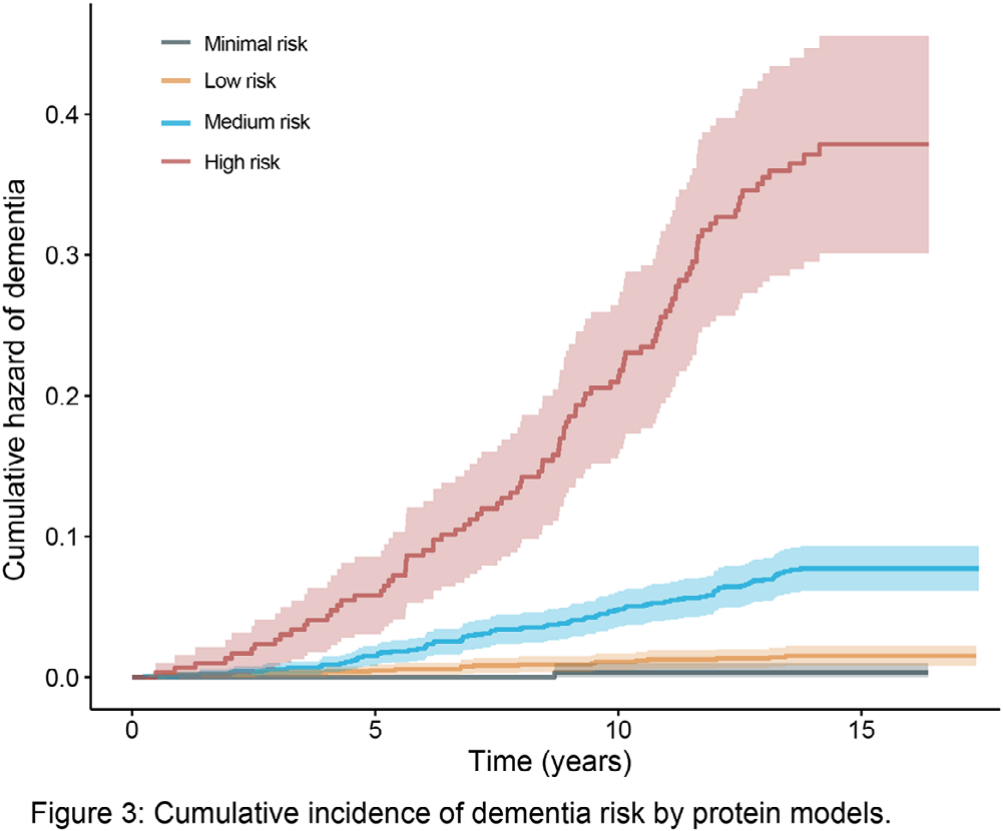# Proteomic dementia risk assessment in adults with diabetes

**DOI:** 10.1002/alz.088890

**Published:** 2025-01-09

**Authors:** Zhibo Wang, Yuye Ning, Pei‐Yang Gao, Meilin Chen, Jianping Jia

**Affiliations:** ^1^ Xuanwu Hospital, Capital Medical University, Beijing, Beijing China; ^2^ Innovation Center for Neurological Disorders, Xuanwu Hospital, Capital Medical University, Beijing, Beijing China

## Abstract

**Background:**

The prevalence of dementia among adults with diabetes represents a significant health challenge. Current dementia risk assessment tools are not tailored to the diabetic population. This study aims to develop more precise dementia risk predictive models for patients with diabetes using extensive blood proteomics.

**Method:**

This prospective cohort study utilized UK Biobank data, encompassing baseline assessments from 2006 to 2010 and follow‐up until September 1, 2023. Excluding individuals with pre‐existing dementia, we included 3015 participants with diabetes and they had available Olink blood proteomics data (approximately 3000 proteins). Feature selection was conducted using a least absolute shrinkage and selection operator (LASSO) regression, and Generalized Linear Models (GLM) were applied to create a proteomic risk model. This model was trained on a tested cohort of 2111 diabetic adults and validated on an additional 904 individuals.

**Result:**

Our model, incorporating 48 proteins, outperformed conventional clinical dementia risk assessment tools, including demographic factors (age, sex, education year, APOE ε4 status) and cognitive assessments. In the validation cohort, the model's receiver operating characteristic area under the curve value was 0.83, significantly higher than the 0.56 to 0.73 range for clinical models (Figure 1). The protein models showed effective calibration in the validation cohort (Figure 2). Participants were divided into four groups based on predicted risk, and the results showed that the higher the level of predicted risk, the greater the risk of dementia (Figure 3). Mendelian randomization suggested a causal link between several proteins and dementia, like apolipoprotein E. Pathway analysis emphasized proteins related to the extracellular matrix, endopeptidase inhibition, and neuron projection.

**Conclusion:**

Our proteomic risk model has high predictive accuracy in identifying dementia risk in adults with diabetes. Because this model uses only blood proteins, it is easier to generalize to the general population and thus easier to guide preventive care in patients with diabetes.